# Efficacy of *Plectranthus amboinicus* (Lour.) Spreng in a Murine Model of Methicillin-Resistant *Staphylococcus aureus* Skin Abscesses

**DOI:** 10.1155/2013/291592

**Published:** 2013-02-20

**Authors:** Francisco Fábio Martins de Oliveira, Alba Fabiola Torres, Thially Braga Gonçalves, Gilvandete Maria Pinheiro Santiago, Cibele Barreto Mano de Carvalho, Milena Braga Aguiar, Lilia Maria Carneiro Camara, Silvia Helena Rabenhorst, Alice Maria Costa Martins, José Telmo Valença Junior, Aparecida Tiemi Nagao-Dias

**Affiliations:** ^1^Department of Clinical Analysis and Toxicology, Faculty of Pharmacy, Universidade Federal do Ceará (UFC), Rua Capitão Francisco Pedro 1210, 60430-370 Fortaleza, CE, Brazil; ^2^Department of Pharmacy, Faculty of Pharmacy, UFC, Rua Capitão Francisco Pedro 1210, 60430-370 Fortaleza, CE, Brazil; ^3^Department of Pathology and Legal Medicine, Faculty of Medicine, UFC, Rua Monsenhor Furtado S/N, 60430-350 Fortaleza, CE, Brazil

## Abstract

The present work aimed to evaluate the effectiveness of *Plectranthus amboinicus* (Lour.) Spreng against MRSA clinical isolates. The in vitro antimicrobial activity of the hydroalcoholic extract (HE), the ethyl acetate (EA) fraction and its subfractions were determined by broth microdilution and bioautography against MRSA clinical isolates. The microdilution checkerboard method was used to assess in vitro drug combination studies. To induce abscess formation, bacterial suspensions were added to Citodex and inoculated subcutaneously into male Swiss mice. The treatment protocol consisted of 2 doses of HE, the EA fraction or vancomycin introduced intraperitoneally into mice 3 and 12 h after infection. The EA fraction and its subfractions presented the lowest minimal inhibitory concentrations (MIC, 0.25 to 0.5 mg/mL). The plant samples were bacteriostatic at 2x and 4x MIC and bactericidal at 100 mg/mL. The EA fraction presented synergism with vancomycin and an additive effect with ciprofloxacin. A significant reduction of abscess volume, bacterial cell counts in abscess slurries, and inflammatory scores was observed in the HE and EA fraction-treated groups. The samples were effective in treating the animals in a dose-dependent fashion. The present study proved the effectiveness of *P. amboinicus* fractions against MRSA using in vitro and in vivo assays.

## 1. Introduction

Recently, methicillin-resistant *Staphylococcus aureus* (MRSA) has gained notoriety as an important human pathogen worldwide. MRSA comprises more than 50% of nosocomial bacterial infections in some countries, including the United States and Brazil [1]. The wide distribution of cases of MRSA in Brazil demonstrates the need for new strategies to improve the control and treatment of these bacterial infections, as their prevalence in health services has reached high levels, ranging from 54% to 93% [2]. MRSA can also affect individuals in the community, thus leading to high morbimortality [3].

Methicillin resistance in staphylococcal infections is due to the acquisition of the mecA gene, which encodes an altered penicillin-binding protein (PBP2a). The 2-kb mecA gene is located on an element of chromosomally inserted DNA that is at least 32 kb and may be greater than 60 kb [4].

Several plants, including *Curcuma longa* [5], *Ducrosia anethifolia* [6], *Rhizophora mucronata* [7], *Acacia aroma* [8], *Zataria multiflora* [9], *Ballota acetabulosa* [10], and *Tylosema esculentum* [11], possess antimicrobial activity against MRSA.

*Plectranthus amboinicus* (Lour.) Spreng (Lamiaceae) is a perennial plant distributed throughout tropical Africa, Asia, Australia, and the Americas, including Brazil [12]. It is popularly known as “hortelã-da-folha-grossa” and “malvarisco.” The species is used in the treatment of several diseases, including skin, digestive, genitourinary, and respiratory disorders, infections, and pain. *P. amboinicus* and *P. barbatus* comprise approximately 68% of the traditional uses of the *Plectranthus* genus [12]. *P. amboinicus* has been a topic of intensive studies because it is rich in metabolites with potential antimicrobial [13–15] and anti-inflammatory activities [16–18]. The leaves of *P. amboinicus* secrete components such as essential oils, flavonoids, and terpenes, which exhibit good antimicrobial activity [14, 15]. 

 Few studies regarding the antimicrobial activity of *P. amboinicus* against *S. aureus* are available. Nogueira et al. [13] first demonstrated the inhibitory effect of the essential oil and hydroalcoholic extract of *P. amboinicus* on clinical strains of methicillin-sensitive *S. aureus* (MSSA). Gurgel et al. [14] demonstrated the bacteriostatic and bactericidal effects of the hydroalcoholic extract of *P. amboinicus* against a standard laboratory strain of MRSA. 

We did not find any studies regarding the antimicrobial activity of *P. amboinicus* in animal models. For this reason, we proposed the present study to investigate the therapeutic effects of extracts from the leaves of *P. amboinicus* in MRSA-infected mice.

## 2. Materials and Methods

### 2.1. Plant Material

The leaves of *P. amboinicus *(Lour.) Spreng were collected from the Horto de Plantas Medicinais Professor Francisco José de Abreu Matos (Fortaleza, Ceará, Brazil). A voucher specimen (EAC40008) was identified by Professor E. P. Nunes and deposited at the Herbário Prisco Bezerra (EAC), Universidade Federal do Ceará, Brazil.

### 2.2. Preparation of the Extracts and Fractions

The hydroalcoholic extract (HE) was obtained by macerating 3 kg of fresh leaves with 70% ethanol (3 litres) for 10 days. The material was concentrated on a rotary evaporator under reduced pressure at 40°C.

The ethanol extract was obtained by macerating 600 g of dried leaves in absolute ethanol for 20 days. The solvent was changed every 5 days. The extract was filtered and evaporated on a rotary evaporator under reduced pressure in a water bath at 40°C. The ethanol extract (80 g) was fractionated by a liquid-liquid partition using a column chromatography (10 × 16 cm) packed with silica gel 60 (70–230 mesh). The elution procedure was performed with solvents of increasing polarities (i.e., hexane, petroleum ether, chloroform, methanol, and ethyl acetate). Solvents were completely evaporated under reduced pressure at 40°C on a rotary evaporator. The fractions were identified as F1 to F5 (Table 1).

A bioassay-guided study of the fractions F1 to F5 was conducted. Because the ethyl acetate fraction F4 presented the best inhibitory effect, it was submitted to a new column chromatography (22 × 6 cm) packed with silica gel 60 (70–230 mesh), and elution was performed with different ratios of hexane and ethyl acetate (Table 2). The subfractions (F4a to F4g) were tested using direct bioautography, and their minimal inhibitory concentrations were determined.

### 2.3. Microbial Strains

The standard control strains used in these experiments were MSSA (ATCC 25923) and MRSA (ATCC 65398), obtained from the Instituto Nacional de Controle de Qualidade em Saúde, Rio de Janeiro, Brazil. Methicillin-resistant strains were isolated from 14 clinical specimens (2 from catheter tips, 10 from blood, and 2 from wound fluid from diabetic foot ulcers) that were biochemically identified by the Clinical Microbiology Department of the Hospital Universitário Walter Cantidio/UFC, Brazil. Screening for the bacterial resistance to antimicrobial drugs was performed using discs (Oxoid, UK) containing trimethoprim/sulfamethoxazole (1.25/23.75 *μ*g), ciprofloxacin (5 *μ*g), gentamycin (10 *μ*g), ampicillin (30 *μ*g), chloramphenicol (10 *μ*g), tetracycline (30 *μ*g), and erythromycin (15 *μ*g) [19]. For vancomycin, bacterial resistance was evaluated using the microbroth dilution assay.

The molecular typing of MRSA was based on the detection of the coagulase gene for species identification and on the presence of the mecA gene confirming methicillin resistance, according to the procedure described by Rallapalli et al. [20]. 

### 2.4. Determination of the Minimal Inhibitory Concentrations (MICs)

To determine the MICs, microdilution tests [21] were performed in 96-well microplates (Costar, USA). The bacterial concentration of standard and clinical isolate strains (1–5 × 10^6^ CFU/mL) was adjusted according to the turbidity of 0.5 McFarland scale. Each well contained 100 *μ*L MHI broth, 5 *μ*L of inoculum, and 100 *μ*L of hydroalcoholic, ethanolic extracts and fractions, and the F4 subfractions, in the range of 0.062–16,000 *μ*g/mL in 5% DMSO. The plates were covered and incubated for 24 h at 35°C.

Vancomycin (Sigma-Aldrich, USA) was used as a positive control, in the range of 0.125–32 *μ*g/mL, and DMSO (Merck, Germany) was used as a negative control, in the range of 0.039–10% v/v. After the incubation period, 10 *μ*L of sterile 0.01% resazurin (Sigma, USA) in aqueous solution was added to the plates. After 2 h of incubation, readings were performed. Resazurin is a blue dye used to visually determine the absence (blue colour) or presence (pink colour) of bacterial growth. 

### 2.5. Bacteriostatic and Bactericidal Activities

Tubes containing 4 mL of MHI broth were inoculated with 1 mL of the MRSA standard strain (ATCC 65398) at a concentration of 10^8^ CFU/mL and were incubated for 2 h at 35°C. A volume of 5 mL of each hydroalcoholic extract or acetyl acetate fraction F4 at 2x or 4x MIC and 100 mg/mL was added to the test tubes, or a volume of 4 mL of BHI broth with 5% DMSO was added to the control tube. The tubes were incubated at 35°C, and aliquots of 30 *μ*L were collected at intervals of 0, 2, 4, 6, 8, and 24 h. Tenfold serial dilutions of the suspensions were plated onto BHI agar to determine a viable cell count. The bactericidal (≥3-log-unit reduction in log 10 CFU/mL) and bacteriostatic activities (<3-log-unit reduction in log 10 CFU/mL) were determined [21].

### 2.6. Checkerboard Method

The microdilution checkerboard method is frequently used to assess in vitro antimicrobial combination studies [22]. The hydroalcoholic extract and the ethyl acetate fraction F4 were tested at 8x MIC. A volume of 100 *μ*L of Mueller-Hinton broth was added to each well of a 96-well microplate. Vancomycin (Sigma, USA) or ciprofloxacin (Miles Inc., USA) was diluted along the horizontal axis, and the plant extracts were diluted along the vertical axis. Five microlitres of the bacterial suspension (MRSA standard strain or 3 MRSA clinical isolates) were adjusted to the 0.5 McFarland standard and added to the plates. After incubation for 24 h at 35°C under aerobic conditions, 10 *μ*L of 0.01% sterile resazurin was added to the plates, followed by 2 h incubation. To evaluate the combinatory effects, fractional inhibitory concentrations (FICs) were calculated. The FIC was defined according to the following formula: the MIC of the drug associated with the plant sample divided by the MIC of the drug or plant tested alone. The sum of the drug's FIC and the plant extract's FIC was considered to be the FIC index (IFIC). The interaction was determined to be synergistic when the IFIC ≤ 0.5, additive when 0.50 < IFIC ≤ 1.0, indifferent if 1 < IFIC < 4, and antagonistic when IFIC ≥ 4.0. The tests were performed in triplicate.

### 2.7. Bioautography

The bioautography technique [23] was employed to evaluate the antimicrobial activity of the subfractions F4a–d. Twenty milligrams of the plant samples were dissolved in 1 mL of methanol and applied in volumes of 10 *μ*L to two glass plates coated with silica gel 60 F_254_. The solvent solution used for elution was hexane: ethyl acetate (6 : 4). After the elution of the components and evaporation of the solvents, a volume of 60 mL of warm overlay agar, previously seeded with a standard MRSA strain (10^6^ CFU/mL), was poured onto the surface of one of the TLC plates. The TLC plate was then placed into a sterile Petri plate and left for 2 h at 4°C and for 24 h at 35°C. The TLC plate was then stained with a solution of tetrazolium bromide dye MTT stain (Sigma-Aldrich, USA) at 5 mg/mL. Yellowish or colourless halos indicated growth inhibition. In parallel, the other TLC plate was submitted to vanillin-perchloric acid staining.

### 2.8. Toxicity Test

For the in vitro cytotoxic activity assay, the macrophage cell line RAW 264.7, obtained from the Rio de Janeiro Cell Bank (BCRJ, Brazil), was grown in microculture plates containing Minimum Essential Media medium supplemented with 10% v/v foetal bovine serum, penicillin (100 U/mL), and streptomycin (100 *μ*g/mL). Cells were incubated at 37°C in 5% CO_2_ and 95% humidity. Before each experiment, the cells were incubated in medium without foetal calf serum for 24 h to obtain cells in the G_0_ phase of the cell cycle. For each experiment, cells were removed from the culture medium and incubated with 0.25% trypsin and 0.02% EDTA (v/v) for approximately 10 min at 37°C. Trypsin was inactivated by adding the same volume of medium containing foetal bovine serum. The suspension was centrifuged for 10 min at 1500 ×g. The supernatant was discarded, and the cells were resuspended in culture medium. The macrophages were quantified using a Neubauer chamber and subcultured (1 × 10^5^ cells/mL) into a 96-well microplate for 24 h. The EH and ethyl acetate fraction F4 at final concentrations of 2x to 4x MICs were added to the microplates. After an incubation of 24 h, 100 *μ*L of the supernatant was discarded and 10 *μ*L of MTT at 500 *μ*g/mL dissolved in phosphate-buffered saline (PBS), mL in phosphate buffered saline, pH 7.4, was added to the wells after incubation for 4 h at 37°C, 10% sodium dodecyl sulphate in 0.01 N HCl was added to solubilise the formazan crystals [24]. Plates were then incubated for 17 h, and readings were performed at 570 nm using a microplate reader. Assays were performed in triplicate.

In vivo toxicity studies of *P. amboinicus *were performed according to the Organization for Economic Cooperation and Development [25]. The protocol proposed by the OECD has advantages over other protocols, as it uses very few animals (*n* = 3) for each step. A single dose of 2000 mg/kg of the HE or the fraction F4 was administered to Swiss mice by gavage. The highest starting dose was used when the test sample was likely to be nontoxic. Animals were observed daily for 14 days. Once no death occurred during the evaluation period, the test was repeated with 3 more animals. No death was reported after the second experiment; therefore, the toxicity of the test sample was categorised as unclassified, and the LD50 was reported to be 5000 mg/kg, according to the OECD [25].

### 2.9. Cutaneous Abscess and Treatment

A mouse skin infection model was established according to the procedure described elsewhere [26]. Animals were housed and cared for in compliance with regional regulations. The experiments were performed in accordance with the protocol approved by the Ethics Committee on Animal Research of the Universidade Federal do Ceara (process 28/09). A clinical isolate (MRSA 08) was grown on a BHI agar plate for 16 h at 35°C. An isolated colony was inoculated into a 5 mL BHI broth tube and left for 6 h at 35°C. Before the experiment, the bacterial suspension turbidity was adjusted to match the 0.5 McFarland standard, diluted to 1 : 10 in phosphate-buffered saline, and added to an equal volume of 2% Cytodex (Sigma, USA). The final suspension (0.2 mL) was inoculated subcutaneously into the back of previously shaved male Swiss mice (25–30 g). The control suspension consisted of BHI broth containing 2% Cytodex. Seven groups of 6 animals in each group were tested. The control group consisted of 4 animals. Two doses of the HE extract, the fraction F4 (250 and 500 mg/kg/dose) or vancomycin (10 and 20 mg/kg/dose) were given intraperitoneally at 3 and 12 h after infection. Abscess diameters were measured after 72 h of infection using a Vernier calliper. The abscess volumes were calculated using the following formula: *V* = (*π*/6)*L* · *W* ^2^ (*L*, length; *W*, width). The animals were then sacrificed by cervical dislocation, and subcutaneous abscesses were excised. After mechanical teasing, abscess slurries were suspended in 0.9% saline and stirred for 10 min. The supernatant was recovered, and tenfold serial diluted aliquots were plated onto BHI agar and incubated for 48 h at 35°C. The number of viable cells was expressed as log CFU/mL. The presence of *S. aureus* was determined by the aspects of bacterial growth, morphology features, and Gram stain characteristics. A small fragment of the injured tissue was removed, fixed in 10% formalin, and submitted for histopathological analysis.

### 2.10. Statistical Analysis

The data underwent analysis of variance (ANOVA) to compare the treatment and control groups, complemented by the Tukey-Kramer test for multiple comparisons. The tests were performed using GraphPad Instat Version 3.01. The level of significance for a null hypothesis was 5% (*P* < 0.05).

## 3. Results and Discussion

The use of medicinal plants has increased in Brazil because they are considered to be a good source for alternative therapeutic purposes. We have recently shown that the essential oil from *P. amboinicus* alters wall permeability and inhibits urease activity and capsule expression of multiresistant strains of *Klebsiella pneumoniae* [15]. The present work aims to show, both in vivo and in vitro, the antimicrobial effectiveness of hydroalcoholic extract and ethyl acetate fraction from *P. amboinicus* against multiresistant *S. aureus*.

Fourteen strains of MRSA isolated from clinical samples of hospitalised patients were identified using biochemical and molecular methods. After screening for oxacillin resistance by disk diffusion, the strains were tested for their susceptibility to other antimicrobial drugs, as shown in Table 3. All strains were resistant to at least 5 antibiotics. The amplification of a 759 bp product by the polymerase chain reaction revealed the coagulase gene, which identified *S. aureus* strains. The amplification of a 533 bp product revealed the mecA gene, which encodes resistance to methicillin. All clinical isolates, except for one (strain 06), contained the mecA gene. Strains with this type of multiresistance are commonly identified in nosocomial infections. When this type of infection occurs, vancomycin or ciprofloxacin is the chosen therapy. In our study, 5 of the 14 strains (35.7%) demonstrated resistance to ciprofloxacin. It is important to be cautious when using either of these antibiotics because vancomycin is nephrotoxic [27], and ciprofloxacin is not indicated for general use in children due to the risk of permanent injury [28]. Our present work demonstrated that all *S. aureus* strains were sensitive to vancomycin. Strains of *S. aureus* with reduced susceptibility to vancomycin have previously been identified by other groups [29].

Table 4 presents the inhibitory activity of the hydroalcoholic and ethanolic extracts and the fractions and subfractions from *P. amboinicus* against MRSA and MSSA standard strains and MRSA clinical isolates. The hydroalcoholic and ethanolic extracts showed activity against the MRSA strains, with MICs varying from 2 to 4 mg/mL and from 4 to 8 mg/mL, respectively. The first report that demonstrated the plant activity against MRSA was published by Gurgel et al. [14]. The authors used the gel diffusion technique to verify that the hydroalcoholic extract of the plant presented MICs of 9.3 mg/mL and 18.6 mg/mL against MSSA and MRSA standard strains, respectively. 

The low activity of the ethanolic extract in our work might be due to the accumulation of substances with no antimicrobial activity, such as proteins, fat, and carbohydrates. For this reason, to isolate the active compounds, the ethanolic extract was subjected to a thin-layer chromatography. The ethyl acetate fraction F4 presented lower MIC values (0.25 to 0.5 mg/mL) than the other fractions and extracts. The antimicrobial activity of the ethanolic extract and its fraction F4 was likely related to the presence of flavonoids and terpenes. These compounds can damage the bacterial cell membrane. According to Hullatti and Bhattacharjee [30], ethanolic extract from *P. amboinicus* presents alkaloid, flavonoids, tannins, triterpenroids, and saponins. In the class of flavonoids, at least quercetin and luteolin were identified [14, 30]. It has recently been demonstrated that both flavonoids exhibit inhibitory activity against MRSA [31].

The fraction F4 was subjected to a new partition chromatography, resulting in seven subfractions called F4a–g. Their MICs are also shown in Table 4. The subfractions F4a–d presented MICs similar to those of the fraction F4 (0.25 to 0.5 mg/mL), except for the subfraction F4a. The MIC of this subfraction varied from 1 to 2 mg/mL.

**Figure 1:**
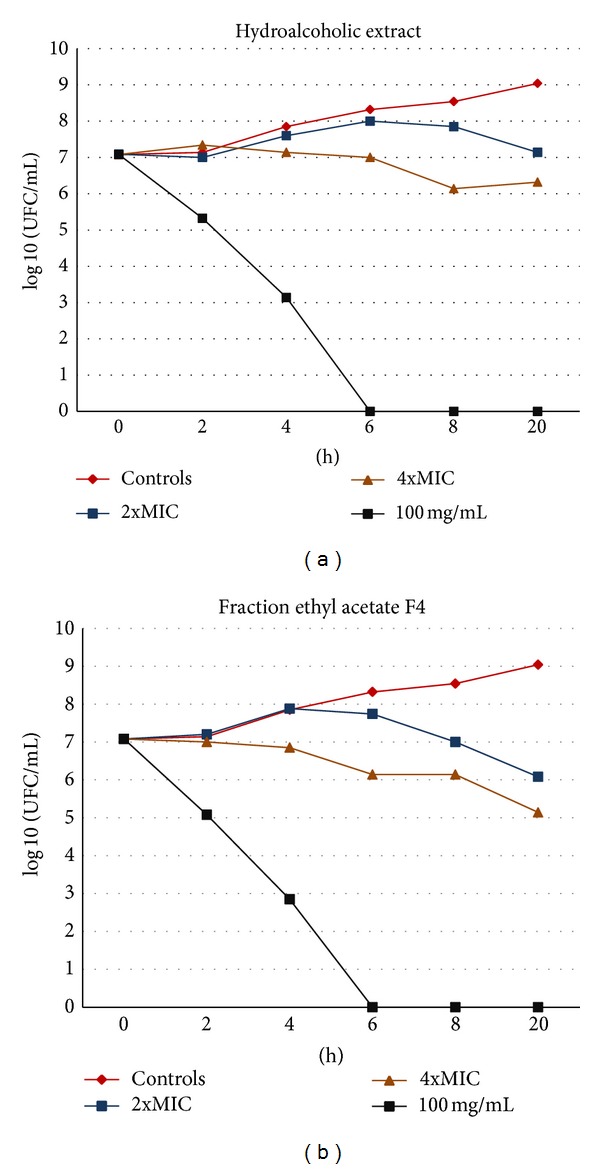
Bacteriostatic and bactericidal activities of the hydroalcoholic (HE) extract (a) and the ethyl acetate fraction F4 (b) from *P. amboinicus* against a standard MRSA strain (ATCC 65398). The results are expressed as log 10 colony-forming units/mL.

The bacteriostatic and bactericidal activities of the hydroalcoholic extract and the ethyl acetate fraction F4 against the standard MRSA strain (ATCC 65398) were evaluated, as shown in Figures 1(a) and 1(b), respectively. The plant samples presented bacteriostatic activity at concentrations of 2x and 4x MIC. Bactericidal activity was observed when the plant samples were tested at 100 mg/mL. Considering the rate and extent of their bactericidal effects, it is necessary to evaluate their minimum bactericidal activity. 

**Figure 2:**
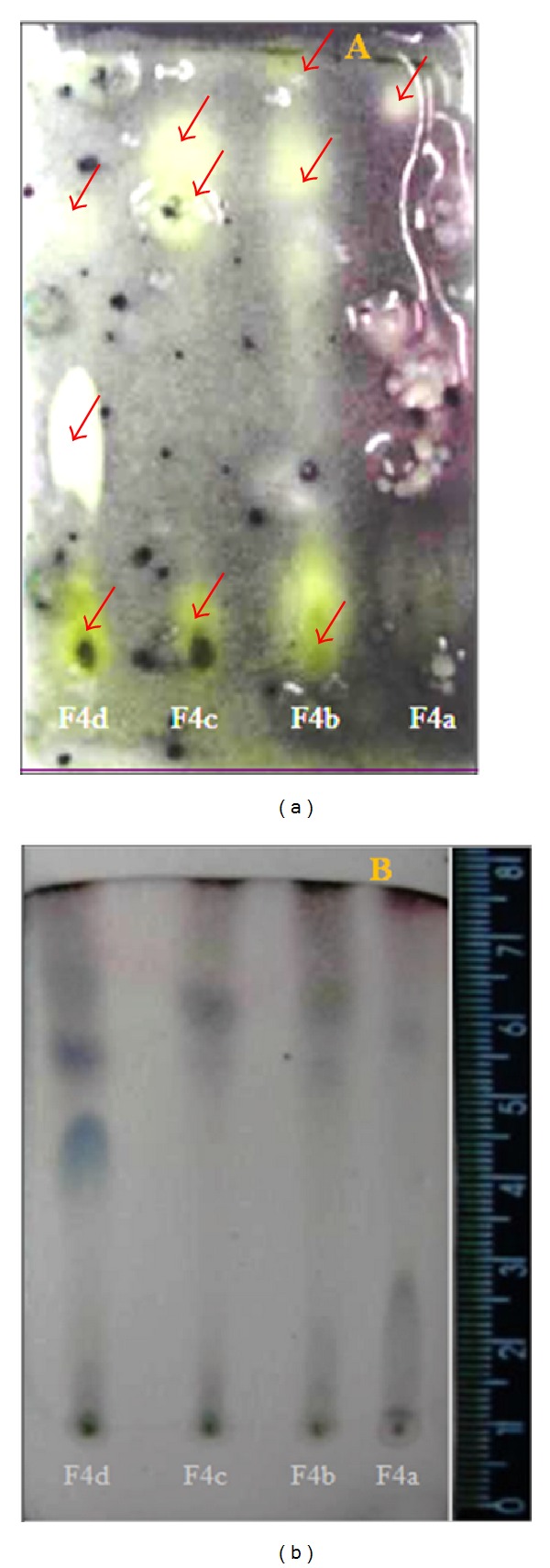
Bioautography of the ethyl acetate F4 subfractions from *P. amboinicus*. Two thin-layer chromatography plates were tested as follows. (a) A developed thin-layer chromatography (TLC) plate was dipped into a Petri dish containing Mueller-Hinton agar previously inoculated with 10^6^ CFU/mL of MRSA. After incubation, the plate was stained with thiazolyl blue tetrazolium MTT stain. (b) A developed TLC plate was directly stained with vanillin/perchloric acid. The arrows indicate the zones of bacterial growth inhibition. The solvent solution used for elution was hexane: ethyl acetate (6 : 4).

The antimicrobial activity of subfractions F4a-d against MRSA was tested using the bioautography technique. Bioautography revealed clear zones representing bacterial zone inhibition (Figure 2(a)). The results suggested that the antimicrobial activity of the subfractions was due to a set of substances with high or intermediate polarities (Figure 2(b)). It is important to remember that the eluting solution contained a mixture of hexane: ethyl acetate (6 : 4), so nonpolar substances and molecules with low polarity would move to the top of the chamber.

A checkerboard method was used to investigate the interactions between the plant samples and the antibiotics (vancomycin and ciprofloxacin). The results are presented in Table 5. The association between the EH extract and antibiotics was considered to be indifferent. Previous reports have shown a synergistic effect of the essential oil from the leaves of *P. amboinicus* with cephalothin and ampicillin as well as antagonistic activity with chloramphenicol and gentamicin [32]. The ethyl acetate fraction F4 showed synergism with vancomycin and an additive effect with ciprofloxacin. These data may suggest that it is possible to associate the fraction F4 with antimicrobials such as vancomycin and ciprofloxacin. The testing combination studies will be conducted in animal models to confirm these in vitro observations.

A slight cytotoxicity was observed when the HE extract and the ethyl acetate fraction F4 were incubated with RAW 264.7 macrophages. Their half-maximal inhibitory concentrations (IC50) were 817.0 *μ*g/mL and 99.6 *μ*g/mL, respectively. Although the HE extract showed a relatively high cytotoxicity, this was not observed with any significant alterations in the in vivo toxicity studies. 

According to Shenoy et al. [33], rats were able to tolerate oral doses of up to 3,000 mg/kg of their body weight of the ethanolic extract of *P. amboinicus* without signs of toxicity. A phase I clinical trial conducted with healthy volunteers [34] to evaluate the safety of a commercial phytotherapic formulation composed of *Schinus terebinthifolius* Raddi, *Plectranthus amboinicus* Lour, and *Eucalyptus globulus* Labill did not demonstrate clinical and laboratory alterations during the period of study. 

Different models of experimental skin and soft-tissue infections have been described and vary widely according to the animal model, bacterial strains, and the period of evaluation. We chose to test in the present work an animal model of subcutaneous abscess using Cytodex with the infecting inoculum [26]. 

**Figure 3:**
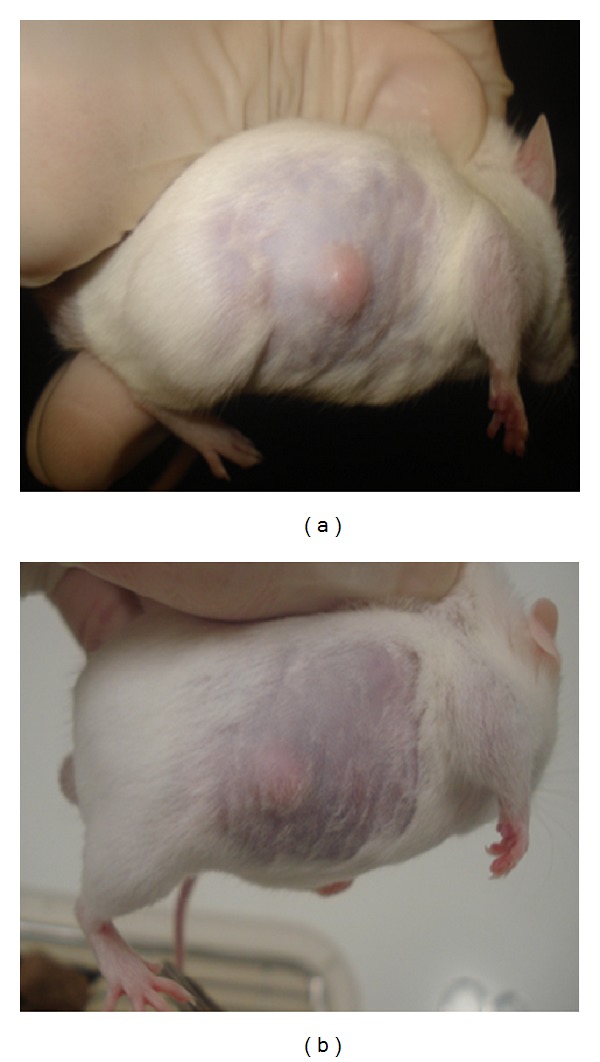
Volume of subcutaneous abscesses in mice infected with an MRSA clinical isolate strain (#08), 72 h after intraperitoneal treatment with 2 doses of ethyl acetate fraction F4 from *P. amboinicus *at 500 mg/kg/dose. (a) Untreated animal. (b) Animal treated with fraction F4.

The samples were effective in treating animals in a dose-dependent fashion. The results are demonstrated in Table 6. The average volume of the abscesses in animals treated with the HE at 500 mg/kg/dose was 63.0 mm^3^, which was significantly lower than those in the animals treated with saline (130.0 mm^3^, *P* < 0.01), but less effective than those in animals treated with vancomycin at 10 mg/kg/dose. There was no difference in abscess volume between the animals treated with the HE at 250 mg/kg/dose and those treated with saline. Abscess volume was significantly reduced in animals treated with the ethyl acetate fraction F4 (Figure 3), both at 500 and 250 *μ*g/kg/dose, in comparison to the untreated animals (*P* < 0.001).

The HE extracts at 500 and 250 mg/kg significantly reduced the number of viable bacterial cells in abscesses compared to the untreated group (*P* < 0.01 and *P* < 0.05, resp.). The fraction F4 was more effective than the HE extract and vancomycin in reducing the bacterial cell counts, compared to the untreated group (*P* < 0.001).

The parameters of inflammation (i.e., cellular infiltration, edema, angiogenesis, fibrosis, necrosis, and haemorrhage) were evaluated and graded on a 0–3 point scale. The sum of the scores was used to estimate the inflammatory status of each animal. Significant improvement in suppurative inflammation was observed in the groups treated with the HE, vancomycin, and the fraction F4, as shown in Table 6. The HE at 500 mg/kg/dose and vancomycin at 20 mg/kg/dose proved to be efficient in resolving the abscesses, compared to the untreated animals (*P* < 0.05). The fraction F4 at 500 mg/kg/dose proved to be the most effective treatment (*P* < 0.001). 

The rapid recovery of the animals treated with the plant samples was most likely due to both the antimicrobial and anti-inflammatory activities of *P. amboinicus*. Some anti-inflammatory effects of *P. amboinicus* extracts are described in the literature, including the blockage of NF-*κ*B activation, which would consequently reduce the production of proinflammatory cytokines [18].

To the best of our knowledge, this is the first report demonstrating the efficacy of *P. amboinicus* in treating a subcutaneous abscess model caused by MRSA. The present study not only corroborates the in vitro model but also demonstrates its efficacy in animal models. This highlights the effective capacity of *P. amboinicus* for use in association with antibiotic therapy for MRSA cutaneous infections.

## Figures and Tables

**Table 1:** Partition of the ethanolic extract of *Plectranthus amboinicus* by using a column chromatography packed with silica gel 60 (70–230 mesh). Elution procedure was done with solvents of increasing polarities.

Eluent	Volume (mL)	Quantity (g)	Fractions
Hex	2000	2.3	F1
Ep	2000	1.2	F2
DCM	5000	35.9	F3
AcOEt	5000	29.6	F4
MeOH	2000	5.9	F5

Hex: hexane; Ep: petroleum ether; DCM: dichloromethane; AcOEt: ethyl acetate; MeOH: methanol.

**Table 2:** Partition of the ethyl acetate fraction F4 of *Plectranthus amboinicus* by using a column chromatography packed with silica gel 60 (70–230 mesh). Elution procedure was done with different ratios of hexane (Hex) and ethyl acetate (AcOEt).

Eluent	Ratio (%)	Volume (mL)	Quantity (g)	Subfractions
Hex : AcOEt	90 : 10	2000	2.1	F4a
Hex : AcOEt	80 : 20	2000	2.4	F4b
Hex : AcOEt	70 : 30	2000	3.2	F4c
Hex : AcOEt	60 : 40	1000	1.5	F4d
Hex : AcOEt	50 : 50	1000	1.1	F4e
AcOEt	100	1000	0.8	F4f
MeOH	100	2000	17.3	F4g

**Table 3:** Resistance of *Staphylococcus aureus* clinical isolates to antimicrobial drugs. The tests were performed according to the CLSI recommendations [19]. The presence or absence of the genes mecA and CoA was verified by the polymerase chain reaction technique, according to the procedure described by Rallapalli et al. [20].

Clinical isolates	Specimen	mecA gene	CoA gene	Antibiotic resistance
Strain 01	BL	+	+	Amp, Chlo, Cip, Ery, Gen, Pen, Sul
Strain 02	BL	+	+	Amp, Chlo, Pen, Sul, Tet
Strain 03	BL	+	+	Amp, Chlo, Cip, Ery, Gen, Pen, Sul, Tet
Strain 04	BL	+	+	Amp, Ery, Gen, Pen, Sul
Strain 05	BL	+	+	Chlo, Ery, Gen, Pen, Tet
Strain 06	BL	−	+	Amp, Chlo, Cip, Ery, Gen, Pen, Sul
Strain 07	BL	+	+	Amp, Ery, Gen, Pen, Sul
Strain 08	BL	+	+	Amp, Chlo, Pen, Sul, Tet
Strain 09	BL	+	+	Amp, Gen, Pen, Sul, Tet
Strain 10	BL	+	+	Amp, Chlo, Ery, Gen, Pen, Sul
Strain 11	CT	+	+	Amp, Chlo, Ery, Gen, Pen, Tet
Strain 12	WF	+	+	Amp, Ery, Gen, Pen, Sul, Tet
Strain 13	CT	+	+	Amp, Chlo, Cip, Ery, Gen, Pen, Sul, Tet
Strain 14	WF	+	+	Amp, Chlo, Cip, Ery, Gen, Pen, Sul, Tet

Catheter tip (CT); blood (BL); wound fluid (WF) from diabetic foot ulcers. Pen: penicillin; Amp: ampicillin; Ery: erythromycin; Cip: ciprofloxacin; Gen: gentamicin; Chlo: chloramphenicol; Tet: tetracycline; Sul: sulfamethoxazole/trimethoprim; Van: vancomycin.

**Table 4:** Minimal inhibitory concentration of extracts, fractions, and subfractions of *P. amboinicus* against MRSA clinical isolates and ATCC.

Strain	Controls			Extracts		Fractions					Subfractions						
	Van (mg/mL)	DMSO (*μ*g/mL)	Bact (% v/v)	EH (mg/mL)	ET (mg/mL)	F1	F2	F3	F4	F5	F4a	F4b	F4c	F4d	F4e	F4f	F4g
ATCC 25923	1	no	+	4	8	no	no	no	0.5	no	2	0.5	0.5	0.5	no	no	no
ATCC 65398	1	no	+	4	4	no	no	no	0.5	no	1	0.5	0.5	0.5	no	no	no
MRSA 01	2	no	+	4	8	no	no	no	0.5	no	1	0.5	0.5	0.5	no	no	no
MRSA 02	2	no	+	2	4	no	no	no	0.5	no	2	0.5	0.5	0.5	no	no	no
MRSA 03	1	no	+	2	8	no	no	no	0.25	no	1	0.5	0.5	0.5	no	no	no
MRSA 04	1	no	+	2	8	no	no	no	0.5	no		
MRSA 05	1	no	+	4	4	no	no	no	0.5	no		
MRSA 06	2	no	+	4	8	no	no	no	0.25	no		
MRSA 07	2	no	+	2	8	no	no	no	0.5	no		
MRSA 08	1	no	+	2	4	no	no	no	0.5	no		
MRSA 09	2	no	+	2	8	no	no	no	0.5	no		
MRSA 10	1	no	+	2	4	no	no	no	0.5	no		
MRSA 11	2	no	+	2	8	no	no	no	0.5	no		
MRSA 12	2	no	+	2	8	no	no	no	0.5	no		
MRSA 13	2	no	+	2	8	no	no	no	0.25	no		
MRSA 14	2	no	+	2	8	no	no	no	0.5	no		

Bact: bacteria; CT: growth control; EH: hydroalcoholic extract; ET: ethanolic extract; F1: hexane fraction; F2: petroleum ether fraction; F3: dichloromethane fraction; F4: ethyl acetate fraction; F5: methanol fraction; F4a–g: subfractions of fraction F4; Van: vancomycin; DMSO: dimethyl sulfoxide; no: no activity.

**Table:** (a) Hydroalcoholic extract (EH)

	CIF		ICIF	Interaction	CIF		IFIC	Interaction
	Eh	Van			Eh	Cip		
ATCC (65398)	1	1	2	I	0.5	2	2.5	I
MRSA 05	1	2	3	I	0.5	2	2.5	I
MRSA 08	1	1	2	I	0.5	2	2.5	I
MRSA 12	1	1	2	I	0.5	2	2.5	I

**Table:** (b) Ethyl acetate fraction (F4)

	CIF		ICIF	Interaction	CIF		IFIC	Interaction
	F4	Van			F4	Cip		
ATCC (65398)	0.5	0.25	0.75	S	0.5	0.5	1	A
MRSA 05	0.5	0.25	0.75	S	0.5	0.5	1	A
MRSA 08	0.5	0.25	0.75	S	0.5	0.5	1	A
MRSA 12	0.5	0.25	0.75	S	0.5	0.5	1	A

CIF: fractional inhibitory concentration; ICIF: fractional inhibitory concentration index.

**Table 6:** Mice infected subcutaneously with an MRSA clinical isolate (strain #08) and treated intra-peritoneally with hydroalcoholic extract (HE) or fraction F4 from *P.  amboinicus* or with vancomycin. The results were expressed by bacterial growth (log colony-forming unities (CFU) per site), abscess volume mean (mm^3^), and by indices of the histopathological analysis.

Treatment	Dosage (mg/kg)	MIC (mg/mL)	Abscess volume mean (mm^3^ ± SD)	*P*	Bacterial cell count Log CFU/site (*x* ± SD)	*P*	Histopathological analysis (index sum ± SD)	*P*
Control			130.0 ± 29.1		7.2 ± 0.41		11.4 ± 0.78	
HE	500	2.0	63.0 ± 26.7	<0.01	5.6 ± 0.51	<0.01	6.7 ± 2.9	<0.01
	250		99 99.5 ± 37.3	N.S.	6.9 ± 0.48	<0.05	9.3 ± 0.82	N.S.
F4	500	0.5	26.9 ± 14.5	<0.001	4.7 ± 0.89	<0.001	5.4 ± 3.1	<0.001
	250		55.1 ± 11.8	<0.001	5.3 ± 0.48	<0.001	6.7 ± 2.7	<0.01
Vancomycin	20	0.2	46.0 ± 22.9	<0.001	4.2 ± 0.91	<0.01	7.5 ± 1.6	<0.01
	10		82.7 ± 27.9	<0.05	5.4 ± 0.86	<0.05	7.8 ± 2.3	<0.05

MIC: minimal inhibitory concentration; SD: standard deviation, N.S.: not significant.

## Abbreviations

BHI: Brain heart infusion
CFU: Colony-forming units
DMSO: Dimethyl sulfoxide
EH: Hydroalcoholic extract
FIC: Fractional inhibitory concentration
FICI: Fractional inhibitory concentration index
MHI: Mueller-Hinton infusion
MIC: Minimal inhibitory concentration
MRSA: Methicillin-resistant *Staphylococcus aureus *
MSSA: Methicillin-sensitive *Staphylococcus aureus *
TLC: Thin-layer chromatography.
